# An update on the arsenal: mining resistance genes for disease management of *Brassica* crops in the genomic era

**DOI:** 10.1038/s41438-020-0257-9

**Published:** 2020-03-15

**Authors:** Honghao Lv, Zhiyuan Fang, Limei Yang, Yangyong Zhang, Yong Wang

**Affiliations:** Institute of Vegetables and Flowers, Chinese Academy of Agricultural Sciences, Key Laboratory of Biology and Genetic Improvement of Horticultural Crops, Ministry of Agriculture, 12# Zhongguancun South Street, Beijing, 100081 China

**Keywords:** Plant breeding, Plant genetics

## Abstract

*Brassica* species include many economically important crops that provide nutrition and health-promoting substances to humans worldwide. However, as with all crops, their production is constantly threatened by emerging viral, bacterial, and fungal diseases, whose incidence has increased in recent years. Traditional methods of control are often costly, present limited effectiveness, and cause environmental damage; instead, the ideal approach is to mine and utilize the resistance genes of the *Brassica* crop hosts themselves. Fortunately, the development of genomics, molecular genetics, and biological techniques enables us to rapidly discover and apply resistance (R) genes. Herein, the R genes identified in *Brassica* crops are summarized, including their mapping and cloning, possible molecular mechanisms, and application in resistance breeding. Future perspectives concerning how to accurately discover additional R gene resources and efficiently utilize these genes in the genomic era are also discussed.

## Introduction

The *Brassica* genus is a member of Brassicaceae (Cruciferae) and contains 39 species (http://www.theplantlist.org/)^1^. Among the *Brassica* species, six constitute U’s Triangle^2^: three diploid species, namely *Brassica rapa* (AA genome: 2*n* = 2× = 20), *Brassica* *nigra* (BB: 2*n* = 2× = 16), and *Brassica* *oleracea* (CC: 2*n* = 2× = 18), and three allotetraploid species, namely *Brassica juncea* (AABB: 2× = 4× = 36), *Brassica napus* (AACC: 2*n* = 4× = 38), and *Brassica carinata* (BBCC: 2*n* = 4× = 34). The triangle model provides the fundamental relationships among these *Brassica* species and is used as an important guideline for both evolutionary research and the improvement of *Brassica* crops via interspecies crossing to facilitate gene exchanges.

**Fig. 1:**
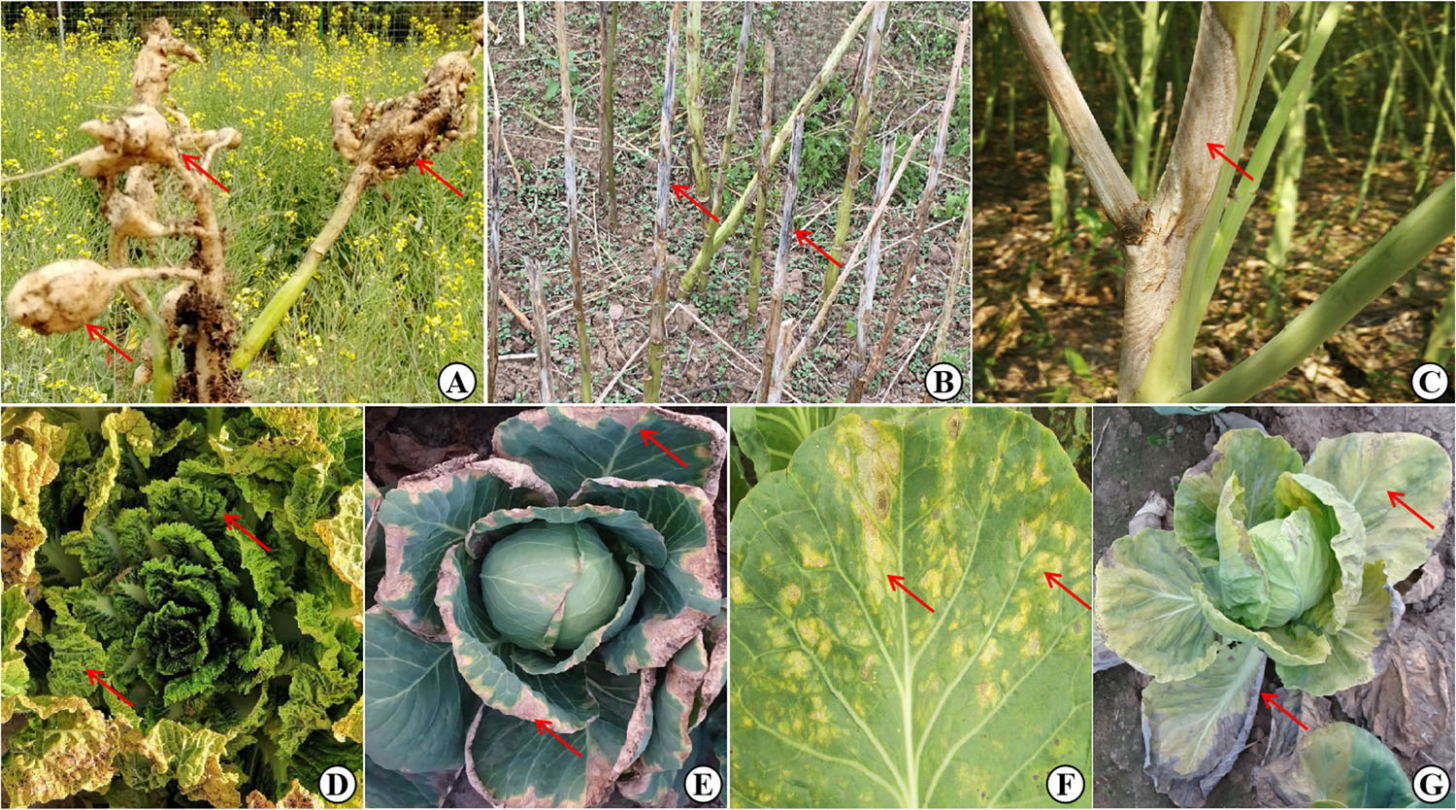
Main diseases in *Brassica* production. **a** Clubroot of *B. napus*. **b** Blackleg of *B. napus*. **c** Stem rot of *B. napus*. **d** TuMV-infected *B. rapa*. **e** Black rot of *B. oleracea*. **f** Downy mildew of *B. rapa*. **g** *Fusarium* wilt of *B. oleracea*. Arrows indicate part of the infected areas with typical symptoms. Images in **a**, **e**, **f**, and **g** were acquired by Honghao Lv in diseased fields in Alberta, Canada, and Hebei, Beijing, and Gansu, China, respectively. Images in **b**, **c**, and **d** were acquired in diseased fields in Hubei, Jiangsu, and Beijing, China, respectively, and were provided by Dr Xiaohui Cheng from the Oil Crops Research Institute, Chinese Academy of Agricultural Sciences (CAAS), Dr Qi Peng from Jiangsu Academy of Agricultural Sciences, and Dr Guoliang Li from the Institute of Vegetables and Flowers, CAAS, respectively

Many *Brassica* crops are of great economic significance, as they are cultivated as vegetables, oilseed sources, condiments, and forages^3^ (Table 1). Climate change, pathogen variation, and inappropriate farming methods, such as continuous and high-intensity cropping, contribute to disease outbreaks, which pose threats to current *Brassica* production. Various pathogens can infect *Brassica* crops and cause production losses, including viruses, bacteria, fungi, and oomycetes (Table 1). Among these diseases, Turnip mosaic virus (TuMV), black rot (BR), blackleg (BL), stem rot (SR), *Fusarium* wilt (FW), downy mildew (DM), and clubroot receive the most attention and are studied most extensively, according to a comprehensive literature search; thus, we will focus on these diseases in the following text (Fig. 1).

**Table 1:** *Brassica* crops and main diseases

Species	Genome	Representative crops	Main diseases
*B. rapa*	AA	Chinese cabbage, turnip, pak choi, caixin	Downy mildew, TuMV, clubroot, soft rot
*B. nigra*	BB	Black mustard	Black rot, leaf spot, blackleg, TuMV
*B. oleracea*	CC	Cabbage, broccoli, cauliflower, kale, brussels sprouts	Black rot, *Fusarium* wilt, clubroot, TuMV
*B. napus*	AACC	Oilseed rape, canola, swede (rutabaga)	Clubroot, blackleg, stem rot, TuMV
*B. juncea*	AABB	Indian mustard, leaf mustard	Blackleg, white rust, stem rot, downy mildew
*B. carinata*	BBCC	Ethiopian mustard	Black rot, TuMV

Traditional approaches for disease prevention include agricultural, physical, chemical, and biological controls, and integrated pest management (IPM) strategies. Physical approaches, such as high-temperature treatment and light trapping, chemicals, such as fungicides and bactericides, and biological agents, such as *Bacillus subtilis* and arbuscular mycorrhizae, are frequently used. IPM has been extensively studied and can achieve some effect for certain diseases. However, the approaches are often complicated, costly, and/or environmentally damaging. In contrast, natural resistance in *Brassica* hosts is the most desirable strategy and could be integrated with other approaches for high-efficiency disease control. Two types of plant immunity have been identified to date: pathogen/microbe-associated molecular pattern (PAMP/MAMP)-triggered immunity, which is activated by cell surface-localized pattern recognition receptors by the recognition of PAMPs/MAMPs, and effector-triggered immunity activated by host resistance (R) genes through the recognition of pathogen-specific effector molecules, which is in accord with the gene-for-gene theory^4,5^. Most R genes identified to date encode nucleotide-binding leucine-rich repeats (NB-LRRs), including coiled-coil NB-LRRs (CC-NB-LRRs) and Toll interleukin 1 receptor NB-LRRs (TIR-NB-LRRs). Moreover, some R genes encode receptor-like kinases (RLKs), transmembrane receptor-like proteins (RLPs), cytoplasmic kinases, and proteins with atypical molecular motifs^6–9^. Various R genes with flexible molecular mechanisms provide powerful weapons that protect the plant host from pathogens.

In recent years, many R genes have been identified and successfully applied to improve *Brassica* crop resistance against various diseases, which not only ensures *Brassica* production but also facilitates the discovery of host–pathogen interactions. Moreover, the genomic era characterized by massive genome and omic data has made fast and accurate R gene studies possible. The release of the reference genome data of the six *Brassica* species in addition to *B. carinata* has provided vital information for determining the genetic and molecular basis of disease resistance^10–14^. Since the 2010s, researchers have performed extensive, high-quality genomic, postgenomic, and omic studies in *Brassica* species and have discovered a variety of R genes and closely related genes, which not only provide further insight into the resistance molecular mechanism and host–pathogen coevolutionary arms race but also facilitate accurate molecular breeding at the whole-genome level.

### Turnip mosaic virus

TuMV is the most prevalent viral disease of *Brassica* crops and causes heavy production losses. In 1921, the disease was first reported in *B. rapa* in the United States^15^, followed by reports in *B. oleracea* in the UK^16^, and in *B. napus* in China^17^. TuMV is currently threatening worldwide *Brassica* crop production, especially in Europe, Asia, and North America, resulting in a production loss of over 30%^18,19^. TuMV exhibits a high level of variation and 12 pathotypes have been revealed thus far, among which 1, 3, and 4 are the most prevalent^20^. The disease is difficult to control due to its rapid variation and nonpersistent mode of transmission by more than 89 aphid species^19,21^. Fortunately, a series of R genes or quantitative trait loci (QTLs) have been characterized and applied in *Brassica* resistance breeding.

Resistance has mostly been uncovered from the A genomes of *B. rapa* and *B. napus*. More than ten TuMV R genes have been characterized in *Brassica* crops thus far (Table 2). *TuRB01*, a monodominant gene for pathotype 1, was first located by Walsh et al.^22^ to a 7.2 cM segment on chromosome N6 of *B. napus*. *TuRB01b* was delimited to a 2.9 Mb segment of A06 from *B. rapa* and comparative analysis showed that *TuRB01* and *TuRB01b* might be similar to each other^23^. *TuRB02*, characterized in the *B. napus* C genome, determines the degree of susceptibility^21^. *TuRB03*, a monodominant gene controlling pathotype 4 resistance, was localized to a 7.9 cM region on N6 in *B. napus*^24^. *retr01* represents the first mapped recessive gene in *Brassica* species^25^; other recessive genes from *B. rapa* include *rnt1* and *trs*, which were mapped to R6 and A4, respectively^26,27^. Using bulked segregant analysis by sequencing (BSA-seq), Shopan et al.^28^ identified another recessive gene, *retr03*, in *B. juncea*. These mapping studies have facilitated the isolation of candidate genes. The dominant gene *ConTR01* and the recessive genes *retr01*, *retr02*, and *retr03* are all assumed to be eIF-encoding genes, whereas *TuRB07*, a monodominant gene from *B. rapa*, was shown to encode a CC-NB-LRR^29^.

**Table 2:** Resistance genes/QTLs identified in *Brassica*

Disease	Species	Pathogen race/isolates	Techniques	Results	Refs.
TuMV	*B. napus*	CHN1, JPN1	RFLP	*TuRB01* in a 7.2 cM interval on N6, *TuRB02* on N14	^22^
	*B. napus*	CDN1	AFLP	*TuRB03* in a 7.9 cM interval on N6	^24^
	*B. rapa*	CDN1	RFLP	*retr01* **and** *ConTR01* **may encode eIF(iso)4E**	^25^
	*B. rapa*	UK1	SSR, InDel	*Rnt1* in a 3.2 cM interval on R6	^26^
	*B. rapa*	C4	Microsatellites, SSR	*retr02* **may encode eIF(iso)4E**	^199^
	*B. rapa*	CHN2, 3, 4, 5	CAPS, SCAR	*trs* tightly linked to *retr02* on A4	^27^
	*B. rapa*	-	SSR, InDel	*TuMV-R* in a 0.34 Mb region on A6	^208^
	*B. rapa*	1	RFLP	*TuRB01b* in a 2.9 Mb region on A6	^23^
	*B. rapa*	C4	BSA, SSR	*TuRB07* **may encode a CC-NB-LRR**	^29^
	*B. rapa*	C4	SSR	*TuRBCS01* in a 1.98-Mb region on A04	^209^
	*B. juncea*	ZJ strains	**BSA, SNP, function analysis**	*retr03* **encodes eIF2Bβ**	^28^
Black rot	*B. oleracea*	-	RFLP	Two major QTLs on LG1 and LG9	^38^
	*B. napus*	4	RFLP	One major QTL (*Xca4*) on N5	^39^
	*B. rapa*	1 and 4	AFLP	Two QTLs for race 1 resistance and four QTLs for race 4 resistance	^40^
	*B. oleracea*	1	EST-SNP	One major QTL (*QTL-1*) on C2	^41^
	*B. oleracea*	1	SSR, CAPS	One major QTL (*XccBo(Reiho)2*) on C8	^42^
	*B. oleracea*	-	RAPD, ISSR, SSR	One major locus (*Xca1bo*) in 1.6 cM interval on C3	^43^
	*B. oleracea*	-	dCAPS	One major QTL on C3	^45^
	*B. carinata*	1	BSA, SSR, ILP	One major locus (*Xca1bc*) in a 6.6 cM interval on B7	^42^
Blackleg	*B. napus*	PG2 isolate PHW1245	RFLP	*LEM1* on A genome linkage group N7	^56^
	*B. napus*	Leroy	RFLP	A major gene, *LmFr1*, and a minor locus	^57^
	*B. nigra*	Four isolates	RAPD	Resistance gene on LG B4	^210^
	*B. napus*	Four isolates	RAPD, RFLP	*LmR1* in A genome linkage group N7	^58^
	*B. juncea*	Isolate 314	RAPD	Resistance gene in LG B8	^211^
	*B. napus*	Field experiment	RAPD, RFLP	Four major genomic regions	^175^
	*B. napus*	Five isolates	RAPD	*Rlm1*, *Rlm3*, *Rlm4*, *Rlm7*, and *Rlm9* in LG10	^60^
	*B. napus*	-	RFLP, SCAR	*LmR1* and *ClmR1* mapped to the same genetic interval in N7	^59^
	*B. juncea*	PG2 isolate	RFLP	*LMJR1* on LG J13 and *LMJR2* in J18	^212^
	*B. rapa*	PG2 and PG3	RFLP	*LepR1* in N2 and *LepR2* and *LepR3*	^66^
	*B. rapa*	31 Isolates	Microsatellite	*LepR3* at an interval of 2.9 cM in LG N10	^67^
	*B. napus*	Isolate 87-41	SRAP	*BLMR1*, with the closet marker of 0.13 cM, and *BLMR2*	^61^
	*B. napus*	Field experiment	SSR	Seven alleles located close to the previous QTLs and five novel alleles	^62^
	*B. napus*	Eleven isolates	SSR, SRAP	14 QTLs, with the major qualitative locus *Rlm4* on chromosome A7	^63^
	*B. napus*	-	NGS, BIA	Several candidates for *Rlm4* on A7	^64^
	*B. napus*	S005, P042 and others	**Function analysis**	*LepR3* **encodes an RLP**	^68^
	*B. napus*	Isolate 165 and others	**Function analysis**	*Rlm2***, an allelic variant of** *LepR3*	^69,70^
	*B. napus*		**Function analysis**	*Rlm9* **encodes an RLK**	^71^
	*B. napus*	Field experiment	SSR	17 QTLs, with six stable ones	^72^
	*B. napus*	WA30 or v23.1.3	DArT	Four QTLs, with a 49 gene QTL interval on chromosome A01	^73^
Stem rot	*B. napus*	-	RFLP, AFLP, SSR	Three QTLs for leaf resistance and three for stem resistance	^81^
	*B. napus*	Isolate 105HT	RFLP	Eight and one QTLs in two DH populations	^82^
	*B. napus*	-	SSR, RAPD, SRAP	Ten, one, and ten QTLs under three different inoculation methods	^175^
	*B. incana*	-	SRAP, SSR	Two major QTLs; 30 candidate genes	^90^
	*B. napus*	SS-1	SSR	**Two major QTLs; one candidate gene (***BnaC.IGMT5.a***)**	^84^
	*B. napus*	-	SSR	Four QTLs for field resistance	^213^
	*B. napus*	-	GWAS	64 Associated genomic regions	^85^
	*B. napus*	-	Comparative genomics	Two genomic regions with conserved QTLs	^214^
	*B. napus*	-	GWAS, SNP array	17 Significant associations on A8 and C6; a candidate GSTU gene cluster	^86^
	*B. napus*	-	GWAS, SNP array	Three associated loci; 39 candidate genes	^87^
	*B. napus*	Isolate #321	GWAS	34 Associated loci	^88^
	*B. napus*	Field isolate	SRAP, SSR	Three common QTLs for different populations	^215^
*Fusarium* wilt	*B. oleracea*	Cong: 1-1 strain	SSR	A linked marker at 1.2 cM	^103^
	*B. oleracea*	FGL3-6, race 1	InDel	*FOC1* in an interval of 1.8 cM	^104^
	*B. oleracea*	FGL3-6, race 1	InDel	**The candidate is a repredicted** *Bol037156*	^105^
	*B. rapa*	Cong: 1-1 strain	RNA-seq	Two candidate R genes identified: *Bra012688* and *Bra012689*	^171^
	*B. oleracea*	Cong: 1-1 strain	SSR	**The candidate is** *Bra012688*	^106^
	*B. oleracea*	FGL3-6, race 1	SRR	A high-efficiency marker located 75 kb from the resistance gene	^207^
Downy mildew	*B. oleracea*	-	RAPD, SCAR	A RAPD marker linked to the resistance gene at 3.3 cM	^116^
	*B. oleracea*	-	RAPD, AFLP, ISSR	*Pp523* in a region of 6.7 cM	^216^
	*B. oleracea*	-	RAPD, SCAR, AFLP	*Pp523* in a region of 4.8 cM	^119^
	*B. oleracea*	-	SSR, SRAP	*BoDM1*, close to a glucosinolate pathway gene	^117^
	*B. rapa*	Beijing isolate	AFLP, RAPD, SSR	The major QTL in a region spanning 2.9 cM	^121^
	*B. rapa*	-	RAPD	*BrRHP1* in a 2.2 Mb interval on A01	^122^
	*B. oleracea*	-	RAPD, ISSR, AFLP	*Pp523* on chromosome C8	^120^
	*B. rapa*	-	SNP, SLAF	*sBrDM8* **may encode a serine/threonine kinase**	^123^
Clubroot	*B. rapa*	Race 2	RFLP, STS	*CRa* in A03	^135^
	*B. rapa*	Race 2 and others	SSR	*Crr1* in A08 and *Crr2* in A01	^142^
	*B. rapa*	Race 2	RAPD	*Crr3* in A03	^144^
	*B. rapa*	Race 2	STS	*Crr3* in a 0.35 cM segment in A03	^145^
	*B. rapa*	Race 4	SCAR	*CRb* in A03	^137^
	*B. rapa*	Race 2 and others	RFLP	*Crr4* in A06	^143^
	*B. rapa*	Race 3	SSR, CAPS	*CRb*^*Kato*^	^140^
	*B. rapa*	Race 2	Mutation analysis	*CRa* **may encode a TIR-NB-LRR**	^136^
	*B. rapa*	Race 3	SSR	*CRb*^*Kato*^, 140 kb interval in A03	^138^
	*B. rapa*	Race 2 and others	**Functional analysis**	*Crr1a* **encodes a TIR-NB-LRR**	^146^
	*B. rapa*	Pathotype 3	SSR, RNA-seq	*Rcr1*, 240 kb interval in A03	^147^
	*B. rapa*	Pathotype 4	BSA, BAC	*CRb*, 83.5 kb interval in A03	^139^
	*B. rapa*	Pathotype 3	KASP, BSR-seq	*Rcr1* in A03, with two candidates	^148^
	*B. rapa*	Five pathotypes	SNP, GBS	*Rcr4* in A03, *Rcr8* in A02, and *Rcr9* in A08	^217^
	*B. rapa*	Five pathotypes	KASP, BSR-seq	*Rcr2* in A03, with two candidates	^149^
	*B. rapa*	Pathotype 3	**Functional analysis**	*CRa* **and** *CRb*^*Kato*^ **are the same allele**	^141^
	*B. oleracea*	Race 7	RFLP	Three QTLs in LG1, LG4, and LG9	^151^
	*B. oleracea*	ECD 16/31//31	RAPD	At least two QTLs	^152^
	*B. oleracea*	Field isolate	RFLP, AFLP	Two QTLs: *pb-3* and *pb-4*	^156^
	*B. oleracea*	Race 1 and 3	RAPD, AFLP	One QTL in LG3	^154^
	*B. oleracea*	P1, P2, P4, and P7	RAPD, RFLP, ACGM	Nine QTLs in 7 LGs	^153^
	*B. oleracea*	Three field isolates	SCAR	Three QTLs in 3 LGs	^155^
	*B. oleracea*	Race 4	SSR, SRAP, SCAR	Five QTLs; the major one is *pb-Bo(Anju)1*	^157^
	*B. oleracea*	Race 2 and 9	GBS	Three QTLs in C2 and C3	^158^
	*B. oleracea*	Race 4	SNP microarray	23 QTLs	^218^
	*B. napus*	Race 2	RFLP	Two QTLs: *CR2a* and *CR2b*	^159^
	*B. napus*	Two isolates	RAPD	One dominant gene (*Pb-Bn1*)	^160^
	*B. napus*	Seven isolates	AFLP, SSR	19 QTLs	^161^
	*B. napus*	Pathotype 3	SSR, InDel	Five QTLs	^162^
	*B. napus*	Pathotype 2, 3, 5, 6, and 8	SSR	A locus in A8 carrying resistance to all five pathotypes	^163^
	*B. napus*	Race 4	Microarray, GWAS	Nine QTLs	^164^

Field experiment indicates natural infection conditions, under which the pathogen stain/race type is usually unclear. The genes in bold represents probable candidates have been found

Molecular markers located close to R loci have been successfully applied in the breeding process through marker-assisted selection (MAS). For example, considering that molecular marker types including amplified fragment length polymorphism (AFLP), random amplified polymorphic DNA (RAPD), and restriction fragment length polymorphism (RFLP) markers present low efficacy and accuracy, Li et al.^30^ designed two Kompetitive Allele-Specific PCR (KASP) markers according to a single-nucleotide polymorphism (SNP) of the TuMV R gene *retr02*, which might be applied in high-throughput MAS. In addition, some resistance genes have been directly applied in resistance breeding. For example, *eIF(iso)4E* variants have been transferred to *B. rapa* and the transgenic plants display broad-spectrum resistance^31^.

### Black rot

BR, the causal agent of which is *Xanthomonas campestris* pv. *campestris* (*Xcc*), is one of the most prevalent bacterial diseases in *Brassica* crops. The disease was first described in the United States by Garman^32^ as a disease of cabbage. The disease has since been identified on all *Brassica*-growing continents, especially in Asia, Europe, and North America, bringing about considerable losses to *Brassica* production^33,34^. *Xcc* exhibits a high level of genetic diversity and 11 races distributed worldwide have been discovered to date, with 1 and 4 being the most prevalent and very virulent to many commercial cultivars^35–37^. In recent years, only a few resistance resources have been identified, greatly hindering the resistance breeding process.

Most BR resistance research conducted to date has focused on QTL analysis or preliminary mapping. The first mapping analysis of BR resistance in cabbage revealed two major QTLs^38^. Vicente et al.^39^ placed a major locus, *Xca4*, in *B. napus* in linkage group N5. Soengas et al.^40^ reported broad-spectrum resistance in *B. rapa*, with a cluster of major-effect QTLs being characterized on A06, each of which could explain 24%–64% of the observed phenotype variation. High-throughput markers allow improved mapping accuracy. Kifuji et al.^41^ applied expressed sequence tag-based SNP markers to map resistance genes in cabbage and three QTLs, including the major *QTL-1*, were detected. Tuno et al.^42^ analyzed BR resistance QTLs and the major QTL *XccBo(Reiho)2* was detected on C8. Saha et al.^43^ mapped the *Xcc* race 1 resistance gene *Xca1bo* in the cauliflower line BR-161 within a 1.6 cM interval. Sharma et al.^44^ first developed a *B. carinata* F_2_ mapping population and mapped the BR race 1 resistance locus *Xca1bc* to a 6.6 cM interval. Lee et al.^45^ first developed genome-wide SNP markers and identified one major QTL on C3 in cabbage. In total, more than 20 QTLs have been placed on over eight *Brassica* chromosomes, indicating that the resistance to BR is highly complicated (Table 2).

Although resistance genes are far from being isolated, some molecular markers closely linked to them have been widely adopted in MAS. Kalia et al.^46^ converted the formerly developed RAPD and inter-simple sequence repeat (ISSR) markers to sequence-characterized amplified region (SCAR) markers, showing great potential for MAS in cauliflower breeding. Using a *B. carinata*-derived F_2_ population, Sharma et al.^44,47^ developed markers linked to BR resistance, which were further used in the selection of introgression lines (ILs) from *B. carinata* to cauliflower.

### Blackleg

BL or stem canker is a disastrous fungal disease for *Brassica* crops caused by *Leptosphaeria maculans* (*Lm*). The first epidemic was reported on cabbage in Wisconsin^48^. However, BL has become a problem in terms of mass infection of oilseed rape only since the middle of the twentieth century, especially in Australia, North America, and Europe^49–51^. *Lm* exhibits a high level of diversification and has been assigned to different races/pathotypes^52–55^. Resistance gene mapping work has been conducted since the 1990s and some cultivars with improved resistance to BL are available.

Most BL resistance genes/QTLs originated from the *B. napus* A genome (Table 2). Ferreira et al.^56^ first applied a double haploid (DH) population from *B. napus* to localize the major locus *LEM1* on N7. Using a similar method, Dion et al.^57^ identified another major gene, *LmFr1*. Mayerhofer et al.^58^ detected a major locus, *LmR1*, and cosegregating markers were developed^59^. Delourme et al.^60^ reported the mapping of resistance loci in two genomic regions and a cluster consisting of five R genes was proposed as the candidate. Fine mapping work was conducted extensively after 2010. Long et al.^61^ identified two resistance genes, *BLMR1* and *BLMR2*, and fine mapping of *BLMR1* resulted in the closest marker distance of 0.13 cM. Jestin et al.^62^ used an association mapping method to characterize the molecular diversity using 128 oilseed rape accessions and identified five novel alleles. Rayman et al.^63^ positioned a new major locus, *Rlm4*, and the deposited region was further analyzed, with several candidates being characterized^64^. In addition, BL resistance loci have been transferred from wild relatives of *B. rapa* and *B. oleracea* to *B. napus*^65^. Yu et al.^66,67^ mapped BL resistance derived from the wild relative and *LepR1*-*LepR3* were identified. Larkan et al.^68,69^ employed map-based cloning to isolate *LepR3*, which encoded an RLP, representing the first cloned BL disease resistance gene; the authors further isolated the *Rlm2* gene, which is an allelic variant of *LepR3*^70^. More recently, the authors cloned another BL resistance gene, *Rlm9*, which encodes a wall-associated kinase-like protein, a newly discovered class of race-specific plant RLK resistance genes^71^. In addition to the major locus, some QTLs have also been characterized, including six and four that are stable under different environmental conditions^72,73^.

Currently, *Brassica* cultivars with improved resistance to BL are frequently cultivated due to extensive R gene mapping work. In addition, MAS is often integrated with other breeding methods to shorten the breeding period. For instance, Yu et al.^65^ described the successful introgression of BL resistance from wild *B. rapa* subsp. *sylvestris* to *B. napus* via interspecific hybridization and MAS, which generates a series of resistant cultivars. In addition, based on both the major genes and QTLs identified, the next breeding effort could involve a combination of qualitative and quantitative loci to provide more durable resistance^74^.

### Stem rot

SR of *Brassica* crops is a fungal disease caused by *Sclerotinia sclerotiorum* (*Ss*). SR is a worldwide catastrophe for *Brassica* production, especially in oilseed rape, in which yield losses can range from 10% to 80%, with low oil quality^75^. *Ss* was reported as the pathogen of SR in 1837 and is now found worldwide^76,77^. *Ss* exhibits little host specificity and eight pathotypes have been identified^78,79^. *Ss* is able to persist for several years in the soil and the most desirable approach for its control to use resistant cultivars^77,80^. Unfortunately, to date, no highly resistant resource has been characterized in *Brassica* crops, making breeding work for SR resistance difficult.

Almost all the mapping work in this context has focused on *B. napus*; however, only partial resistance has been characterized in both the A and C genomes (Table 2). Zhao and Meng^81^ first identified three QTLs for leaf resistance and three other QTLs for stem resistance in the seedling and adult stages, respectively, but no common QTLs. Zhao et al.^82^ identified eight and one QTL involved in two segregating DH populations, with each explaining 6–22% of the observed variance, still with no common QTLs. Yin et al.^83^ detected ten, one, and ten QTLs in one DH population using three inoculation procedures, and only two common QTLs were detected. Wu et al.^84^ identified three QTLs at the seedling stage for leaf resistance and ten QTLs for stem resistance at the adult stage. Two major QTLs could be detected repeatedly and a candidate resistance gene, *BnaC.IGMT5*, was first identified. These studies revealed abundant QTLs but seldom common ones, indicating the complicated genetic structure of these plants. Recently, the release of the *B. napus* genome sequence has strongly facilitated mapping work. Fomeju et al.^85^ first adopted a genome-wide association study (GWAS) using 116 materials genotyped with 3228 SNPs and the results indicated that 64 genomic regions are involved in SR resistance. Wei et al.^86^ combined GWAS and SNP array analyses using 347 *B. napus* accessions and 17 significant regions were located on the A8 and C6 chromosomes. These SNPs on Chr. A8 were placed in a 409 kb segment, with candidate genes being suggested. Using a similar method, Wu et al.^87^ genotyped 448 accessions and 26 SNPs corresponding to three loci were associated with SR resistance. In total, 39 candidates were proposed. Gyawali et al.^88^ performed a GWAS using microsatellite markers in a global collection of 152 accessions and found that 34 loci were significantly associated. To date, many loci opposing SR have been characterized but none have been functionally characterized.

Considering that high resistance to SR in *B. napus* is not available, researchers tend to investigate wild *Brassica* relatives for novel germplasm, such as *Berteroa incana* and *Brassica cretica*. MAS combined with distant hybridization plays significant roles in resistance transfer. For example, Mei et al.^89–91^ successfully introgressed resistance from wild *B. incana* into *B. napus* through hexaploidy hybridization and MAS using newly developed simple sequence repeat (SSR) markers and phenotype evaluation.

### *Fusarium* wilt

FW disease, caused by the fungus *Fusarium oxysporum* f. sp. *conglutinans* (*Foc*), is posing a threat to *Brassica* production worldwide, especially for cole crops^92,93^. FW was first observed on cabbage by Smith^94^ in the United States in 1895. Since 1910, FW has spread quickly from the United States to almost the whole world^95,96^. To date, two *Foc* races have been reported, but only race 1 is found worldwide^97–99^. FW is a soil-borne disease and *Foc* can survive for more than 10 years, even without a host^100^. Currently, type A resistance conferred by a dominant monogene has been identified and applied successfully. However, race 2 can overcome type A resistance, indicating that single resistance application is at high risk.

Most resistance resources have been identified in *B. oleracea* (Table 2). Specifically, two types of resistance have been characterized, i.e., A and B. Type A resistance is stable under high or low temperature and follows a single dominant inheritance pattern; type B polygenic resistance is unstable under high temperatures above 24 °C^101,102^. The type A single dominant resistance gene for *Foc* race 1 has been explored extensively in the last several years. The FW R gene *FocBo1* was first mapped to linkage group seven using both BSA and QTL analysis by Pu et al.^103^. Lv et al.^104,105^ generated a genetic linkage map based on a cabbage DH population and mapped the R gene *FOC1* to a 1.8 cM interval between two adjacent InDel markers. The authors further mapped the candidate gene *FOC1* to a repredicted *Bol037156*, which encodes a TIR-NBS-LRR, using an enlarged population. Shimizu et al.^106^ also mapped the resistance locus *FocBo1* by using 139 recombinant F_2_ plants and identified a candidate gene, *Bra012688*. The two mapped candidates are homologous with high identity. However, the functions of these genes remain to be identified.

Type A resistance to *Foc* race 1 conferred by a dominant single gene, *FOC1*, has been successfully mapped and molecular markers have been developed and applied to generate various resistant cultivars. In addition, MAS using these markers has been combined with other breeding methods to promote the breeding process. For example, Lv et al.^107^ reported the use of isolated microspore cultures with MAS to rapidly obtain target DH lines with FW resistance, which could be used directly in resistance breeding, thereby shortening the breeding period by 2–3 years.

### Downy mildew

DM is a foliar disease of *Brassica* crops and the causal agent is the oomycete pathogen *Hyaloperonospora brassicae* (*Hb*)^108,109^. DM causes considerable yield losses to all *Brassica* crops worldwide, especially in Europe, Asia, and Australia^110,111^. Physiological races or pathotype variations have also been described in various studies; however, few studies have achieved clear race differentiation^112–114^. Notably, Coelho et al.^115^ summarized six pathotypes and suggested five major-effect R loci corresponding to the observed phenotypes. The ideal control approach for DM is to breed genetically resistant varieties. To date, several R loci have been mapped and applied in breeding.

Resistance to DM is thought to be distinct at the *Brassica* seedlings and adult stages. Resistance mapping work has identified several R genes/loci (Table 2). In *B. oleracea*, the first locus conferring resistance in the broccoli seedling stage was placed in a linkage group^116^ and was found to be located close to the glucosinolate-related gene *BoGsl elong*^117^. Another single dominant resistance gene expressed at the adult stage was identified in broccoli and was named *Pp523*^118^. The genomic region containing this gene was further analyzed using SCAR and cleaved amplified polymorphic sequence (CAPS) markers, as well as two bacterial artificial chromosome (BAC) libraries^119,120^. In *B. rapa*, QTLs conferring seedling-stage resistance were discovered. Using a genetic linkage map generated with a DH population, the major-effect locus *BraDM* was delimited to a region spanning 2.9 cM in the A08 linkage group^121^. For adult-stage resistance, a monodominant gene, *BrRHP1*, was localized to a 2.2 Mb interval on the A01 linkage group^122^. In recent years, mapping methods based on high-throughput resequencing have greatly promoted the identification of R genes. For example, using a high-density SNP-based map, a major locus, *sBrDM8*, was localized to a physical segment of ~228 kb, with one candidate kinase gene, *Bra016457*^123^.

The developed markers closely located with these R loci have been adopted for resistance breeding through MAS and have greatly contributed to resistance breeding. For example, Yu et al.^124^ converted the closely linked RAPD marker K14-1030 to a SCAR marker, which greatly improved selection efficiency in the progenies.

### Clubroot

Clubroot (CR) caused by *Plasmodiophora brassicae* (*Pb*) is now threatening almost all *Brassica* crops worldwide. *Pb* is neither a fungus nor a slime mold and has been classified into the new taxon Rhizaria^125^. CR was first reported in Russia in 1878^126^ and rapidly expanded to Europe, Asia, and America, becoming one of the most serious problems for *Brassica* production around the world^125,127^. *Pb* exhibits complex pathotypes and two differentiation systems are used extensively: the Williams system and the European clubroot differential set^128–131^. The variation in this pathogen and its ability to survive in soil in the form of resting spores make it difficult to control^132–134^. Thus, breeding resistant cultivars represents an ideal control method. Currently, extensive studies addressing CR have generated the largest number of resistance loci among all *Brassica* diseases (Table 2) and MAS has been widely used for resistance improvement.

In *B. rapa*, several important CR genes conferring complete resistance in accessions against specific pathogen isolates have been identified. The mapping and cloning of the *CRb*/*CRa* loci took over 20 years. *CRa* was mapped and the candidate gene encodes a TIR-NBS-LRR^135,136^. Another locus, *CRb*, from the Chinese cabbage cultivar CR Shinki, was extensively mapped to a final 84 kb region^137–139^. Kato et al.^140^ identified another CR resistance locus, *CRb*^*Kato*^, in Akiriso Chinese cabbage. Hatakeyama et al.^141^ further determined that *CRb*^*Kato*^ and *CRa* were the same TIR-NB-LRR gene, whereas *CRb* might be a different locus. Another example is the *Crr1-4* genes from turnip, which were initially primarily mapped using different molecular markers and populations^142–145^. Through fine mapping, Hatakeyama et al.^146^ discovered that *Crr1* consists of two genes: *Crr1a* and *Crr1b*. The former encodes a TIR-NB-LRR and was functionally confirmed. With the development of genomic and molecular genetics, several loci were further identified using newly developed marker techniques^147^. Yu et al.^148^ applied BSA-seq and identified a novel resistance gene, *Rcr1*, and two candidates encoding TIR-NB-LRRs. Huang et al.^149^ adopted KASP markers and BSR-seq strategies to finely map *Rcr2* to a 0.4 cM interval, identifying two TIR-NBS-LRRs as candidates. Using BSA-seq, Pang et al.^150^ identified the new locus *CRd* in a 60 kb region on chromosome A03, which is located upstream of *Crr3*.

In *B. oleracea*, CR resistance appears to be determined by quantitative genes. Figdore et al.^151^ first identified three QTLs conferring resistance to *Pb* race 7 in broccoli. In the resistant kale line C10, Grandclément and Thomas^152^ performed QTL detection with RAPD markers and the results indicated at least two types of genetic mechanisms. Rocherieus et al.^153^ further found two to five QTLs depending on which of five pathotypes were used and *Pb-Bo1* was uncovered for all *Pb* isolates, accounting for 20.7–80.7% of the phenotypic variation. In another resistant kale line, K269, Moriguchi et al.^154^ and Nomura et al.^155^ detected two and three loci, respectively, conferring resistance to different isolates. In cabbage, Voorrips et al.^156^ first reported two major QTLs, *pb-3* and *pb-4*. Nagaoka et al.^157^ identified a major QTL, *pbBo(Anju)1*, from the cabbage accession Anju. Lee et al.^158^ employed the genotyping by sequenceing (GBS) technique and a QTL survey to reveal two and one major loci for races 2 and 9, respectively. These loci showed positions close to the previously identified resistance loci in *B. oleracea* but in distinct locations from those discovered in *B. rapa*, indicating divergence of R loci between the *Brassica* A and C genomes.

For *B. napus*, a few loci conferring resistance to various isolates have been characterized. Landry et al.^159^ identified two QTLs controlling CR resistance to race 2, which contributed 58% and 15% of the observed phenotypic variation. Manzanares-Dauleux et al.^160^ reported the mapping of R loci in Darmor-bzh and identified one major gene, *Pb-Bn1*. Using a DH population, Werner et al.^161^ detected 19 QTLs that conferred resistance to 7 different isolates, but none of them could confer resistance to all these isolates. Fredua-Agyeman and Rahman^162^ mapped canola CR resistance to a DNA segment that comprised 12 markers linked to the *CRa* locus, indicating its possible A genome origin. Hasan and Rahman^163^ used rutabaga-derived populations for resistance mapping and characterized a genomic segment on chromosome A8 conferring resistance to all five tested pathotypes. GWAS enables rapid detection of recombinants and variations using natural populations based on whole-genome SNP data. Li et al.^164^ first applied GWAS to 472 accessions to identify CR resistance with the 60 K *Brassica* Infinium SNP. A total of nine loci were characterized through integrative analysis, with seven of them being novel and six of them being in the C genome.

The closely linked markers and resistance genes have been widely used in *Brassica* CR resistance breeding, generating a series of resistant cultivars that successfully control CR in many areas. For example, considering that high resistance is found mostly in *B. rapa*, researchers have frequently applied interspecies crossing to facilitate R gene transfer combined with MAS and phenotype evaluation^165,166^.

## Future perspectives

### Creating novel germplasms via close or distant hybridization

For a certain *Brassica* species, the resistance resources for diseases such as BR, BL, SR, and CR are highly limited. In general, the A genome is rich in TuMV, BL, DM, and CR resistance, whereas the B genome possesses BR and BL resistance, and the C genome harbors SR, FW, and DM resistance. Fortunately, the six species in the *Brassica* genus and others, such as *B. incana*, *B. cretica* (C genome), and *Brassica fruticulosa* (B genome), as well as its close Brassicae relatives, could be used to facilitate resistance gene exchanges in breeding programs.

Interspecies crossing within the *Brassica* genus is widely adopted using embryo rescue, reciprocal crossing and MAS. For example, there are notably few BR-resistant resources in the C genome of *B. oleracea*, whereas high resistance is present in both the A and B genomes. Thus, interspecies hybridization has been used to transfer and utilize the resistance found in the A and B genomes. Tonguç and Griffiths^167^ developed interspecific hybrids between *B. oleracea* and *B. juncea* accession A19182, which show resistance to both *Xcc* races 1 and 4. The progenies displayed resistance to both races. Similarly, Sharma et al.^47^ successfully transferred BR resistance from *B. carinata* to cauliflower. In addition, distant hybridization has been used by breeders to generate novel resistant germplasms. For example, only partial resistance to BL has been characterized in *B. napus* thus far, whereas complete or highly resistant lines are not available. In contrast, high-level resistance was observed in a few wild species, such as *Erucastrum cardaminoides*, *Diplotaxis tenuisiliqua*, and *Sinapis arvensis*. Snowdon et al.^168^ performed a cross between *B. napus* and *S. arvensis*, and through resistance tests and molecular analyses, the ILs were successfully identified from the BC_3_ progenies, which exhibited high resistance at both the seedling and adult stages. Garg et al.^169^ first obtained high levels of SR resistance from crosses between *B. napus*/*B. juncea* and *E. cardaminoides*/*D. tenuisiliqua*/*Erucastrum abyssinicum*. The novel resistance germplasms generated in such studies provide valuable materials in future breeding programs for *Brassica* crops. However, there is still much work to be done for them to be practically applied in commercial cultivars. For example, there are rich resources with CR resistance in *B. rapa* but very few in *B. oleracea*, and breeders have spent ~17 years introgressing resistance from Chinese cabbage (*B. rapa* subsp. *pekinensis*) cv. Parkin to different *B. oleracea* cultivars, which are now widely used^170^.

### MAS as an approach for high-efficiency integrated breeding in the genomic era

Molecular markers are inheritable and detectable genomic segments. The techniques for molecular markers and gene mapping have been significantly improved from the 1990s to the present in the genomic era. First, methods such as RAPD, AFLP, and RFLP, representing the first-generation markers with low efficiency, were constantly applied to map the resistance genes and QTLs for CR, BR, BL, and TuMV. Since the 2000s, convenient and highly efficient markers, including SSRs, microsatellites, and InDels, have gradually become mainstream and have been applied for the discovery of novel R loci for SR, CR, and BL. As the 2010s, mapping methods based on high-throughput sequencing data have developed rapidly, especially SNP-based methods, such as KASP markers and microarrays, BSA/BSR and GWAS. Based on whole-genome level mutations and their association with trait values, GWAS enables fast and accurate target trait gene characterization using natural populations. For example, Wei et al.^86^ applied combined GWAS and SNP array analyses to 347 *B. napus* accessions to detect resistance to SR and identified 17 significant associations on two chromosomes. These SNPs on chromosome A8 were localized to a segment of 409 kb, with candidate genes being proposed. In addition, KASP technology possesses high assay robustness and accuracy, and allows notable savings in terms of cost and time. Huang et al.^149^ adopted KASP markers and BSR-seq strategies to rapidly identify the *Rcr2* locus in the CR-resistant Chinese cabbage cultivar Jazz and *Rcr2* was delimited to a 0.4 cM region, where two TIR-NBS-LRRs were identified as candidates. In addition, based on the sequence variations of the TuMV resistance gene *retr02*, Li et al.^30^ designed a KASP marker that could be used to accurately genotype the allele in Chinese cabbage accessions.

In many cases, there is one main disease in a specific production region and application of cultivars with resistance to that main disease is appropriate to realize the balance between defense and growth. However, for some continuous or high-intensity cropping regions, various pathogens may accumulate; therefore, cultivars with multiple resistances to different diseases are in great need. Currently, MAS using abundant molecular markers enables us to realize the pyramiding of R alleles for different diseases and breed multiresistant cultivars. MAS combined with other methods, such as hybridization and microspore culture, has greatly shortened the breeding circle. These cultivars are now available on the market, such as cabbage cv. Zhonggan 628 (Institute of Vegetables and Flowers, Chinese Academy of Agricultural Sciences, Beijing, China), with resistance to TuMV and FW; Chinese cabbage cv. Jingchun CR1 (Beijing Academy of Agriculture and Forestry Sciences, Beijing, China), with resistance to TuMV, DM, and clubroot; and oilseed rape cv. Huashuang 5R (Huazhong Agricultural University, Wuhan, Hubei, China), with resistance to TuMV and clubroot, and tolerance to SR.

**Fig. 2:**
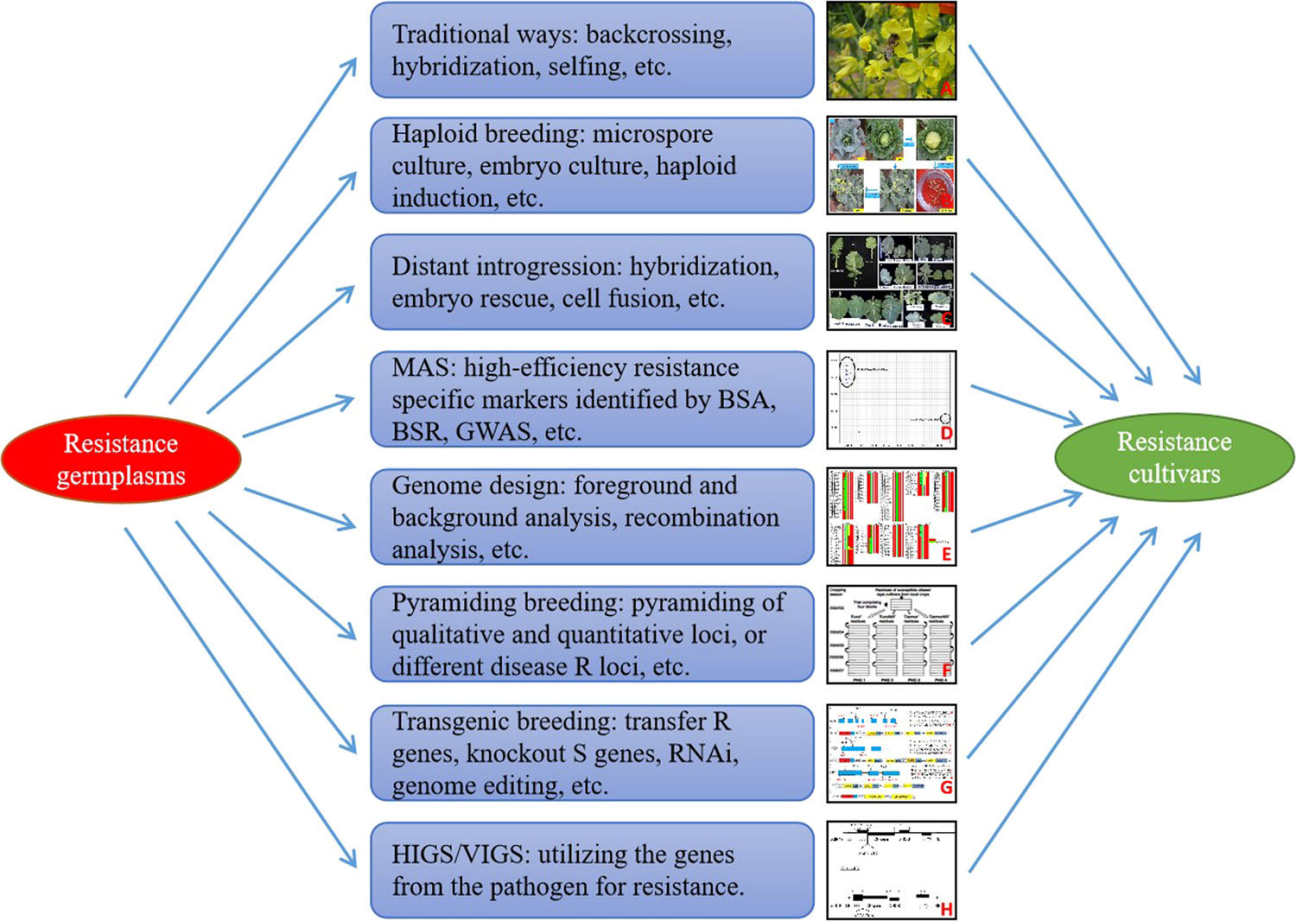
Proposed high-efficiency integrated breeding (HIB) model in the genomic era. **a** Selfing using honeybees is one of the most traditional breeding methods. **b** A combined use of microspore culture and MAS helps promote the breeding cycle in *B. oleracea*^107^. **c** BR resistance introgression from *B. carinata* to *B. oleracea* using distant hybridizing and embryo rescue^47^. **d** SNP-based high-throughput KASP markers prove efficient and cost saving in genotyping during MAS in *B. rapa*^30^. **e** Whole-genome background analysis helps eliminate the undesired linkage drags during MAS in *B. oleracea*^207^. **f** Pyramiding both the qualitative and quantitative R loci generates durable BL resistance in *B. napus*^74^. **g** CRISPR/Cas9-based gene editing helps knockout multiple target genes in *B. oleracea*^172^. **h** Expressing the *CP* gene from TuMV confers high resistance in *B. napus*^200^

The genomic era is also characterized by high-efficiency integrated breeding (HIB), in which multiple methods are combined, including traditional ways, such as microspore culture, backcrossing, and distant introgression, and modern ways, such as MAS, gene editing, and genome design (Fig. 2). During HIB, genomic background analysis is helpful in eliminating undesirable linkage drags and rapidly identifying desirable individuals. For example, in a study by Liu et al.^171^, resistance-specific markers and genome background markers were used to breed cabbage with resistance to FW. By combining these methods with microspore culture and backcrossing, the authors presented a rapid and effective approach for generating FW-resistant ILs in the BC_2_ generation. Notably, the quickly emerging gene-editing technique helps realize accurate alteration of the target DNA sequence. Ma et al.^172^ applied CRISPR/Cas9-mediated multiple gene editing in cabbage, with the targets *BoPDS*, *BoSRK*, and *BoMS1*, and successfully generated albino, self-compatible, and male sterile lines, showing its great power in improving plant traits.

### Pyramiding of qualitative and quantitative loci to acquire durable resistance

A single resistance gene is easily overcome because of pathogen variations and global climate changes. For example, a few *B. rapa*, *B. oleracea*, and *B. napus* varieties resistant to specific *Pb* races have been successfully cultivated. However, all these varieties lose resistance within a few years. At the same time, vast genetic variability in the clubroot pathogen *Pb* and infection by multiple races have been reported^129–131^. For the BR pathogen *Xcc*, pathogen variations are frequently discovered and at least 11 races have been reported thus far^35–37^.

More durable resistance is urgently needed to ensure *Brassica* crop production. Durable resistance was first proposed by Johnson^173^ as resistance that maintains effectiveness during long-term widespread application. Complete race-specific resistance genes are very effective in a short period but are easily overcome by the pathogens; polygene-inherited resistance is thought to be more durable, but its effects might be unstable owing to variable environmental conditions^174^. Thus, pyramiding qualitative genes with major quantitative loci in cultivars represents an ideal means to ensure the effectiveness and durability of resistance. An example is the utilization of BL resistance in *B. napus*. Brun et al.^74^ evaluated a cultivar with single race-specific *Rlm6*-mediated resistance and another cultivar with both *Rlm6* and quantitative resistance in a 5-year field experiment. The single *Rlm6* resistance became ineffective as soon as the third cropping season. When integrated with quantitative resistance, however, *Rlm6*-mediated resistance maintained effectiveness until the seventh year. Another cultivar, Jet Neuf, has been widely used for as long as 10–15 years, both as a cultivar and as a source of resistance. Jet Neuf was shown to harbor both polygenic resistance and the mono R gene *Rlm4*^50,175,176^. This pyramiding model is also supported and used in resistance breeding against BR^39^, SR^177^, and CR^127^. Thus, combining quantitative resistance with single R genes is a promising strategy for resistance breeding. In addition, coevolution between the host and the pathogen has been extensively studied; e.g., the mapped seven BL resistance genes/alleles and their contrasting avirulence genes have indicated a rapid and fierce arms race between *Brassica* hosts and *Lm*, and the single use of one type of pyramiding could pose a high selection pressure on the pathogen and thus raise the risk of pathogen mutation and host resistance loss. Thus, the rationalized pyramiding of dissimilar sets of resistance genes/QTLs should be deployed in different cultivars to provide heterogeneity in the selection pressure on the pathogen population and result in more durable resistance^178^. In addition, pyramided resistance could be integrated with agricultural, physical, chemical, and biological controls to realize IPM, further maximize durability and guarantee stability.

### Digging deeper into the resistance mechanism in prebreeding studies

To date, hundreds of R loci in *Brassica* crops have been characterized; however, candidates have only been found for approximately a dozen of them and their mechanisms are far from being revealed compared with those of the model plants *Arabidopsis*, tobacco, and rice. Current molecular and omics methods, including transcriptomics, proteomics, and metabolics, provide new opportunities for mining genes in the resistance-regulating network, which could be either directly used in resistance breeding or used in indirect prebreeding studies to promote our understanding of *Brassica*–pathogen interactions. For example, in SR, transcriptomic and proteomic studies have revealed a series of key genes associated with the response to pathogen infection, including RLKs, NBS-LRRs, calcium-binding proteins, PRs, TFs, and polygalacturonase inhibitor proteins, which are associated with plant–pathogen interactions, the mitogen-activated protein kinase signaling cascade, plant hormone biosynthesis and signaling, and oxalic acid (OA) metabolism^179–184^. These genes were subjected to functional clarification and prebreeding studies. For instance, overexpression of *BnMPK4*^185^, *BnWRKY33*^186^, chimeric chitinase^187^, *OA*^188^, and *PGIP2*^189^ can enhance host resistance. In addition, the sequenced *Brassica* accessions do not contain all R genes due to variations between individuals, whereas the establishment of the pangenomes could facilitate gene mining from a wider platform. Bayer et al.^190^ performed a comparative analysis of resistance gene analogs (RGAs) in the pangenome of *B. oleracea* and identified 59 RGAs linked to SR, CR, and FW resistance, some of which probably resulted from a recombination within R gene clusters.

**Fig. 3:**
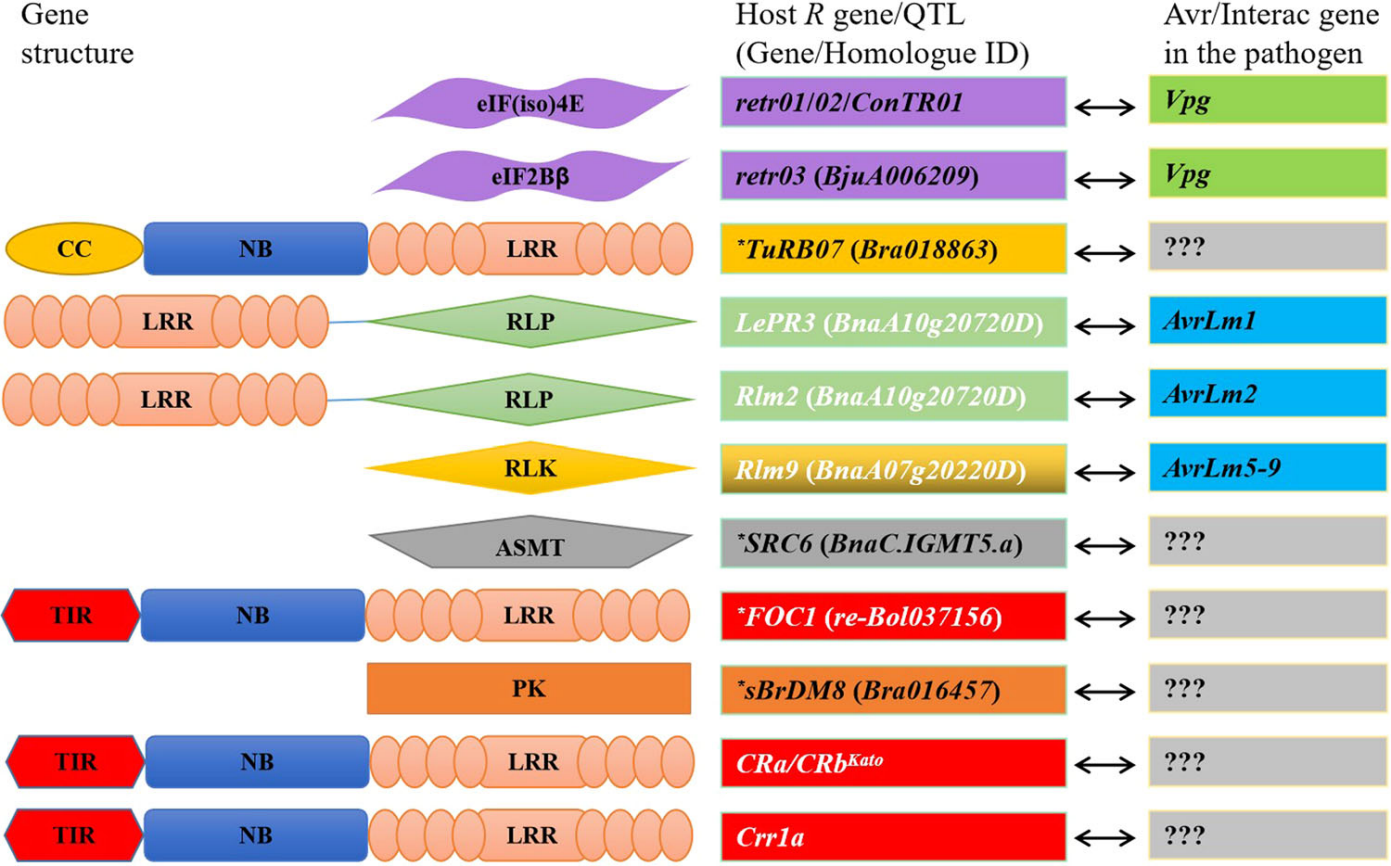
Resistance genes identified in *Brassica* crops and their avirulence/interactor genes in the pathogens. ASMT, N-acetylserotonin *O*-methyltransferase; CC, coiled-coil domain; eIF(iso)4E, eukaryotic translation initiation factor isoform 4E; eIF2Bβ, eukaryotic translation initiation factor 2Bβ; LRR, leucine-rich repeat; NB, nucleotide-binding domain; PK, protein kinase; RLP, receptor-like protein. *Putative genes that have not been functionally validated. ???The avirulence or interaction genes in the pathogens that have not yet been characterized

Achieving expanded knowledge of the molecular host–pathogen interactions could provide evidence for disease control. Protein–protein interactions in the *Brassica*-TuMV system are a heavily researched topic. Previous studies have shown that the cytoplasmic inclusion protein determines the viral avirulence for TuRB01/01b/04, whereas P3 determines the avirulence of TuRB03/05^191–194^. Another example is the plant eukaryotic initiation factor 4E (eIF4E) family, which is well characterized as a key factor during the invasion of several potyviruses. The viral protein genome-linked (VPg) protein of potyviruses interacts directly with the host eIF4E/eIF(iso)4E and determines virulence^195,196^. This eIF4E-mediated mode of resistance is generally strong and broad spectrum^197,198^. In *Brassica*, the recessive R genes to TuMV, including *retr01*, *retr02*, and *trs*, have been identified to encode eIF(iso)4E^25,27,199^. Furthermore, researchers induced different key amino acid mutations in eIF(iso)4E by a systematic knowledge-based approach to interrupt the interaction between TuMV VPg and host eIF(iso)4E, and transgenic plants with eIF(iso)4E variants display high and broad-spectrum resistance^31^. This example shows the great potential of artificially designed R alleles/mutants in resistance breeding. In addition to the direct application of the identified resistance genes, genes from TuMV have also been used in resistance breeding as a method of host-induced gene silencing, especially the *CP* gene. The CP protein can accumulate in host cells and inhibit virus replication, thereby conferring resistance. Successful resistance enhancement via the *CP* gene strategy has been reported in *Brassica* crops, including oilseed rape and Chinese cabbage^200,201^. In *Brassica* crops, although over ten R genes have been characterized to date, most avirulence or interaction genes in the pathogens have not yet been thoroughly characterized (Fig. 3). Thus, the next emphasis should be on the establishment of global collections of pathogen isolates for the identification of important avirulence or interaction genes. In addition, for the host, the development of a series of ILs through recurrent backcrossing to “Mendalize” the quantitative loci would be beneficial. For example, to clarify the relationship between the *Lm* isolates and the mapped seven BL resistance genes/loci, Larken et al.^202^ introgressed each of the seven R genes/loci into a common susceptible *B. napus* DH line through reciprocal backcrossing, producing single R gene ILs that could provide for the accurate assessment of Avr-R gene interactions by avoiding non-Avr-dependent alterations.

To date, using the *Arabidopsis*–*Brassica* pathogen pathosystem, great progress has been made in the characterization of resistance genes and their molecular mechanisms, which can provide critical clues for *Brassica* resistance studies, as both *Brassica* and *Arabidopsis* belong to Cruciferae. For example, the interactions between *Hb* and *Arabidopsis* have been well established. To date, more than 10 *RPP* loci conferring resistance to *Hb* have been cloned in *Arabidopsis*, most of them being NLRs that regulate the activation of programmed cell death^203,204^. In addition, some important genes, such as *EDS1*, *NDR1*, *PRs*, *NPRs*, and *WRKYs*, have been shown to play important roles in DM resistance in *Arabidopsis*^205^. In addition, the pathogen effectors have been isolated and the host–pathogen interactions have also been well characterized^206^. As many R genes have been cloned in *Arabidopsis*, the orthologous genes in *Brassica* can be investigated, which will greatly facilitate the cloning of these genes and the clarification of their molecular functions.

## Concluding remarks

*Brassica* species comprise many economically important crops, but their production is constantly threatened by emerging diseases, such as TuMV, BR, FW, DM, and clubroot. The most ideal measure is to mine and utilize the resistance genes of the *Brassica* crop hosts themselves. Fortunately, the development of genomics, molecular genetics, and biological techniques enables us to rapidly discover more than 100 R genes/loci. However, only a dozen of them have strong candidates and are still not well functionally validated. Moreover, only a small portion of them has been applied in resistance breeding. Thus, the next efforts should be more accurate identification of the R genes and clarification of their molecular mechanisms using emerging high-efficiency genomic, postgenomic, and omic methods, and more efficient application of the R resources through an integration of approaches, such as haploid culture, MAS, distant introgression, genome design, pyramiding, and transgenic breeding, to control the diseases and secure *Brassica* production.
